# Removal of Chromium(VI) by Nanoscale Zero-Valent Iron Supported on Melamine Carbon Foam

**DOI:** 10.3390/nano12111866

**Published:** 2022-05-30

**Authors:** Qiming Li, Meili Liu, Xuchun Qiu, Xiang Liu, Malcom Frimpong Dapaah, Qijian Niu, Liang Cheng

**Affiliations:** 1 School of Environment and Safety Engineering, Jiangsu University, Zhenjiang 212013, China; jingtian19941106@163.com (Q.L.); liumeili202110525@163.com (M.L.); xuchunqiu@gmail.com (X.Q.); 5103200301@stmail.ujs.edu.cn (M.F.D.); 2 Institute of Medicine & Chemical Engineering, Zhenjiang College, Zhenjiang 212000, China; liuxiang0222@126.com; 3 School of Agricultural Engineering, Jiangsu University, Zhenjiang 212013, China; 4 School of Civil and Mechanical Engineering, Curtin University, Perth, WA 6102, Australia; 5 Institute of Materials Engineering, Nanjing University, Nantong 226000, China

**Keywords:** melamine foam, nano zero-valent iron, Cr(VI) removal, safety evaluation

## Abstract

The overuse of chromium (Cr) has significantly negatively impacted human life and environmental sustainability. Recently, the employment of nano zero-valent iron (nZVI) for Cr(VI) removal is becoming an emerging approach. In this study, carbonized melamine foam-supported nZVI composites, prepared by a simple impregnation–carbonization–reduction method, were assessed for efficient Cr(VI) removal. The prepared composites were characterized by XPS, SEM, TEM, BET and XRD. Batch experiments at different conditions revealed that the amount of iron added, the temperature of carbonization and the initial Cr(VI) concentration were critical factors. Fe@MF-12.5-800 exhibited the highest removal efficiency of 99% Cr(VI) (10 mg/L) at neutral pH among the carbonized melamine foam-supported nZVI composites. Its iron particles were effectively soldered onto the carbonaceous surfaces within the pore networks. Moreover, Fe@MF-12.5-800 demonstrated remarkable stability (60%, 7 days) in an open environment compared with nZVI particles.

## 1. Introduction

The quest for rapid economic development has increased chromium (Cr) discharge into the environment due to the expansion of notably textile, leather tanning and metal-based industries [1,2]. These discharged Cr heavy metals exist dominantly in trivalent (Cr(III)) and hexavalent (Cr(VI)) forms, with the latter being more hazardous due to their strong toxicity and mobility [3], causing severe health problems such as multiorgan failure, renal necrosis and pulmonary fibrosis [4]. Hence, Cr(VI) standards for industrial effluents (0.1–0.5 mg/L) and drinking water (<50 µg/L) have been stipulated by the U.S. Environmental Protection Agency (USEPA) to curb water contamination [5]. Till date, broad categories of techniques were developed for Cr(VI) removal, including electrocoagulation [6], adsorption [7], bioremediation [8] and chemical reduction [9], of which adsorption (and/or subsequent reduction) is much preferred as it is easily designed, effective, tractable, economically feasible and no secondary contamination [10].

In comparison to conventional adsorbents (e.g., titanium dioxide, goethite, zeolites), nano zero-valent iron (nZVI) has the advantages of higher reactivity and surface energy, more active surface sites, and higher reaction rate [11,12]. However, the aggregation of nZVI limits its mobility, dispersity, durability and, mechanical strength, and the oxidization of nZVI can significantly decrease its reactivity [13,14]. Additionally, nZVI and its end products are toxic and can cause pollution if not well managed. Its interaction with biological species and promotion of disruptive oxidant generation heightens ecotoxicity [4,15,16]. To overcome these disadvantages, nZVI is often loaded onto supporting materials, including sepiolite, mesoporous silica, resin, and biochar [17,18,19,20]. Considering high stability, large specific surface area and, excellent adsorption performance, carbon-containing materials such as activated carbon, graphene and, carbon nanotubes are primarily utilized to support nZVI [21,22,23,24,25,26]. Nonetheless, these commonly used carbon-based materials have the disadvantages of high cost and complex preparation procedures.

Cheap commercial melamine foams consist of three-dimensional (3D) interlinked dendritic fibers with high elasticity, large geometric area, and apertures of about 4 microns [27]. There have also been reports showing potential applications of melamine foams. Wu et al. reported a microporous metalloporphyrin-containing framework-wrapped melamine foam for process-intensified acyl transfer [28]. Additionally, Li et al. developed superelastic and arbitrary-shaped graphene aerogels with a sacrificial skeleton of melamine foam [29]. Furthermore, Rodon Fores et al. prepared a catalytically active supramolecular hydrogel, using commercial melamine foam, that was used in a continuous flow reactor with good recyclability [30]. Melamine foam can also be carbonized to form a honeycomb-type microporous structure with high nitrogen and oxygen co-doping by a simple high-temperature carbonization process [31]. This type of material exhibits excellent adsorption capacity (>100 times their weight), high porosity (>99%), and low density (~7 mg/cm^3^) [32]. In addition, it displays good chemical stability and electrical conductivity, which can effectively facilitate electron transfer [33]. Although carbonized melamine foam possesses the above advantages, there is still no research on carbonized melamine foam loaded with nZVI for Cr(VI) removal.

In this work, carbonized melamine foam loaded with nZVI (denoted as Fe@MF) was prepared and optimized using a simple one-stage carbon thermal reduction method. The morphology and characteristics of Fe@MF were investigated by X-ray photoelectron spectroscopy (XPS), X-ray diffraction (XRD), transmission electron microscopy (TEM), specific surface area (BET), and scanning electron microscopy (SEM). Additionally, the application of this material in Cr-containing sewage treatment was studied.

## 2. Materials and Methods

### 2.1. Chemicals

Potassium dichromate (K_2_Cr_2_O_7_), iron (III) chloride hexahydrate (FeCl_3_·6H_2_O) (99%), sodium borohydride solution (NaBH_4_), sulfuric acid (H_2_SO_4_), sodium hydroxide (NaOH) and hydrochloric acid (HCl) were purchased from Sinopharm Chemical Reagent Co. Ltd. (Shanghai, China). Deionized (DI) water was used in all tests. The Cr(VI) stock solution (100 mg/L) was prepared by dissolving K_2_Cr_2_O_7_ in DI water. Different concentrations of Cr(VI) solution (10–60 mg/L) were obtained by diluting the stock with DI water.

### 2.2. Fe@MF Composite Preparation

The nZVI-loaded carbonized melamine foam composite was prepared following the procedure of impregnation–carbonization–reduction described earlier [23] (Figure S1a). Firstly, 20 mL of FeCl_3_·6H_2_O solution with concentration ranging from 7.5 to 50 mmol/L was added to melamine foam (SINOYQX) blocks (5 × 3 × 2 cm^3^). The porous melamine foam soaked with 0, 7.5, 10, 12.5, 25, 37.5, and 50 mmol/L FeCl_3_·6H_2_O solutions were named Fe@MF-0, Fe@MF-7.5, Fe@MF-10, Fe@MF-12.5, Fe@MF-25, Fe@MF-37.5 and, Fe@MF-50, respectively. Secondly, the wet melamine foam blocks were dried at 50 °C for 48 h, followed by calcination in a tube furnace at 800 °C (10 °C/min heating rate) under N_2_ atmosphere for 1 h. The obtained composites were labeled Fe@MF-0-800, Fe@MF-7.5-800, Fe@MF-10-800, Fe@MF-12.5-800, Fe@MF-25-800, Fe@MF-37.5-800, and Fe@MF-50-800, respectively. For Fe@MF-12.5, different carbonization temperatures of 600 °C, 800 °C, and 1000 °C were applied and subsequently labeled Fe@MF-12.5-600, Fe@MF-12.5-800, and Fe@MF-12.5-1000.

After carbonization, the carbonized melamine foam showed a perforated network structure similar to a tetrapod architecture [34]. Although, after carbonization, the volume of the samples decreased by about two times, the 3D porous structure was still maintained (Figure S1b–d).

### 2.3. Analysis

According to the methods described by Milacic et al., Cr(VI) concentration was assessed with 1,5-diphenylcarbazide utilizing a spectrophotometer (UV-3101PC, Sakaemachi, Japan) at 540 nm [35]. Morphologies of the synthesized composites were analyzed using SEM equipment (Hitachi SU8020, Tokyo, Japan). The sample elemental compositions were examined with an EDS (energy dispersive X-ray spectroscopy) detector (Horiba Emax 7593-H, Tokyo, Japan) attached to the SEM. Additionally, TEM images were obtained with a JEOL JEM-2100F TEM equipment (200 kV accelerating voltage: 0.23 nm point-to-point resolution). The oxidation states of elements on Fe@MF-12.5-800 surface before and after Cr(VI) removal were obtained by an X-ray photoelectron spectrometer (Shimadzu, Axis, Sakaemachi, Japan). The C 1s charge correction has been set to 284.8 eV (from 285.0 eV). The background was fitted using Avantage V5.52. The line shape was also fitted by Avantage V5.52 and the %Lorentzian–Gaussian was 20%. X-ray diffraction (XRD) patterns for phase and crystallite analysis were collected (5–90°, 2°/min) on a Bruker D8 Advance (Karlsruhe, Germany) at room temperature. Nitrogen adsorption–desorption of Fe@MF-12.5-800 was determined on an Autosorb iQ-MP Quantachrome instrument (Boynton Beach, FA, USA). Fe content was analyzed using an inductively coupled plasma optical emission spectrometer (Agilent technologies 700 Series ICP OES, Palo Alto, CA, USA).

### 2.4. Batch Experiments

The performance of Fe@MF composites under various conditions, including Fe^3+^ concentration (7.5 to 50 mmol/L) and the temperature of carbonization (600 °C, 800 °C, and 1000 °C), were conducted via batch experiments. The stability of the produced composites was investigated by measuring Cr(VI) removal efficiency after the Fe@MF composites were kept in an open environment for a desired period (0 to 7 days). Unless specified elsewhere, all the experiments were conducted in stirring flasks containing 10 mg/L Cr(VI) solution at pH 7. Additionally, all experiments stated above were conducted at room temperature.

### 2.5. Column Trial

Fe@MF-12.5-800 was used as the filling material in a column reactor for flow-through wastewater treatment. In this test, 10 mg/L Cr(VI) solution was taken as feedwater. The flow rates were set at 1 and 2 mL/min. For each treatment cycle (360 mL/cycle), the effluent was collected and measured for its Cr(VI) concentration. The removal efficiency was calculated according to the initial (influent) and final (effluent) Cr(VI) concentrations shown in the following Equation (1):

(1) $$\mathrm{Removal}\;\mathrm{capacity}\;\left(µg\right)=\left(C_{0}-C_{e}\right)\times V\times 1000$$

where *C*_0_ and *C_e_* denote the initial and final Cr(VI) concentrations (mg/L) per cycle and *V* represents the Cr(VI) solution volume (L) per cycle [4].

## 3. Results and Discussion

### 3.1. Optimization of Fe@MF Synthesis

**Figure 1:**
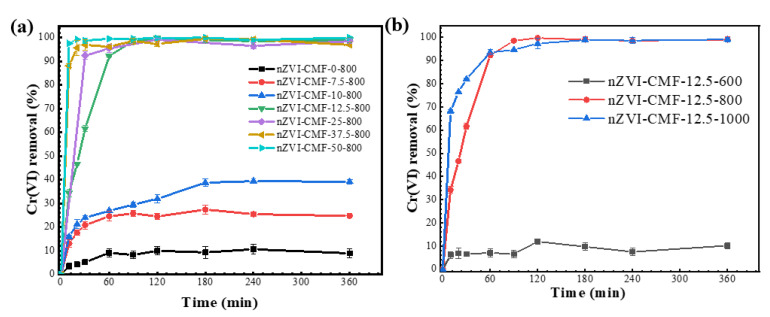
Cr(VI) elimination by Fe@MF with (**a**) different concentrations of Fe^3+^ loaded and (**b**) different temperatures of carbonization applied.

From Figure 1a, Cr(VI) elimination was significantly affected by the Fe^3+^ concentration present in the melamine foam. Overall, its removal rate upsurged with increasing Fe^3+^ amounts. At Fe^3+^ concentration higher than 12.5 mmol/L, a rapid Cr(VI) adsorption of >99% occurred within 90 min. The removal efficiency significantly decreased from 99.75% to ≤39.13% when a smaller amount of Fe^3+^ (<12.5 mmol/L) was used. The control sample of carbonized melamine foam could remove about 10% Cr(VI) probably due to the porous structure formed during the carbonization, which was in line with previous findings [26]. By considering both the economic aspect and removal efficacy, the 12.5 mmol/L was selected as the optimum concentration.

It is well known that the carbonization temperature could remarkably affect nZVI formation [36]. The sample carbonized at 600 °C indicated a rather low Cr(VI) removal of about 12% (Figure 1b), similar to that of the control sample without Fe^3+^, suggesting that nZVI could not be formed at this temperature. It has been reported that Fe^3+^ reduction to Fe^0^ requires a specific temperature and sufficient carbon. Generally, Fe_3_O_4_ tends to form at relatively low temperatures (e.g., 500 °C), whereas Fe^0^ amounts increase gradually with temperature [37,38]. The samples produced at 800 °C and 1000 °C displayed significant Cr(VI) removal efficiency with a slightly greater initial removal rate at the higher temperature. From the perspective of energy consumption, 800 °C was selected as the optimum carbonization temperature.

### 3.2. Characterization of Fe@MF-12.5-800

**Figure 2:**
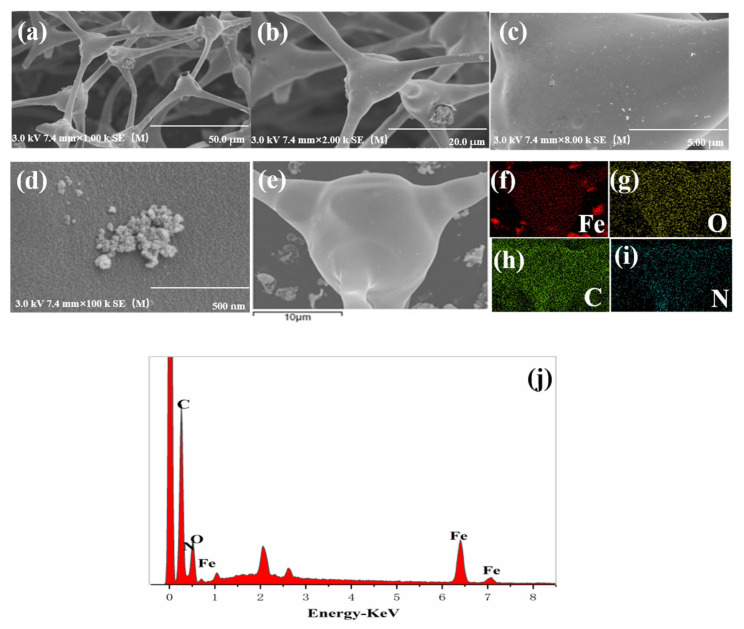
Characteristics: SEM images of (**a**) Fe@MF-12.5-800 (×1000), (**b**) Fe@MF-12.5-800 (×2000), (**c**) Fe@MF-12.5-800 (×8000) and (**d**) Fe@MF-12.5-800 (×100,000). (**f**–**i**) SEM-EDS elemental distribution mapping of Fe, O, N and C obtained from (**e**). (**j**) The EDS survey of Fe@MF-12.5-800.

Figure 2a shows that the morphology of Fe@MF-12.5-800 has a well-developed porous structure. Such an arrangement would be conducive to the penetration of the solution, thus increasing the contact area between the solution and the material for better pollutant removal [24]. It can be seen that nanoparticles with irregular spherical shapes were observed on the microfiber surfaces of the carbonized melamine foam (Figure 2b,c). These nanoparticles were mildly aggregated (Figure 2c,d) compared with the previously described behavior of pure nZVI particles. This suggests that the Fe@MF composite produced using the proposed method is beneficial for nZVI dispersion. EDS element mapping of the Fe@MF-12.5-800 clearly shows the presence of carbon, nitrogen, oxygen, and iron (Figure 2e–j). The iron was from FeCl_3_·6H_2_O, while nitrogen originated from melamine foam and N_2_ gas used during the carbonization [29]. The presence of nitrogen functional groups could provide active sites in the composite and improve adsorption capacity [39]. Overall, the EDS analysis revealed that the melamine foam was successfully doped with iron particles after carbonization and the Fe content measured by ICP-OES was 13.26%, similar with that in EDS and XPS (Table S1).

The N_2_ adsorption/desorption experiment shows that the BET surface area of Fe@MF-12.5-800 was 303.29 m^2^/g with a pore volume (PV) of 0.28 cm^3^/g. The obvious hysteresis between the durative increase in the adsorption capacity and desorption curves before P/P_0_ = 0.4 reveals the coexistence of mesopores on Fe@MF-12.5-800 (Figure S2a). The pore size distributions (as shown in Figure S2b) further confirmed its mesopore structure with various pore sizes mainly ranging between 2 and 10 nm. This well-developed mesoporous structure implies its potential to be a high-performance adsorbent.

**Figure 3:**
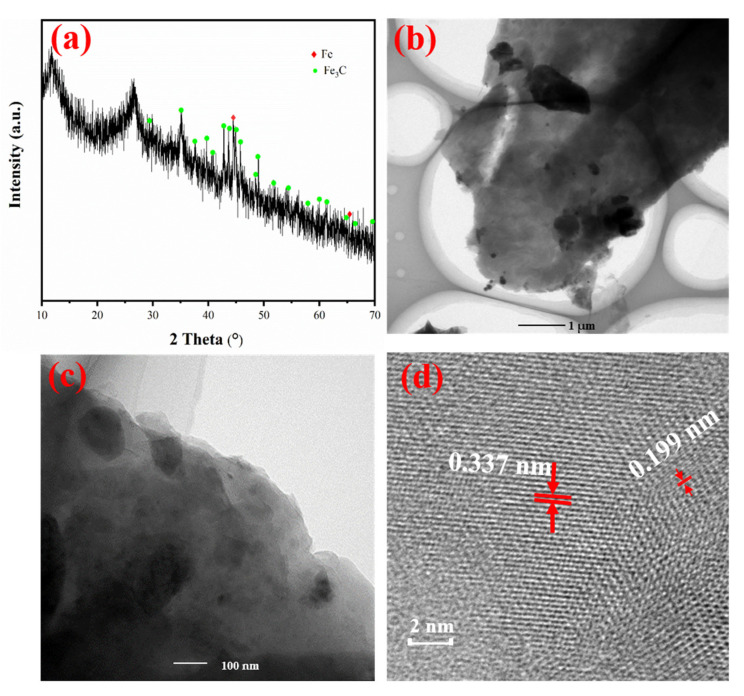
(**a**) X-ray diffraction patterns of Fe@MF-12.5-800; (**b**,**c**) TEM images and their respective particle size distribution of Fe@MF-12.5-800; and (**d**) HRTEM image of Fe@MF- 12.5-800.

The XRD spectrum of Fe@MF-12.5-800 (Figure 3a) evidenced its crystallographic structure. Those distinct peaks occurring at 44.8° and 65.1° corresponded to the Bragg plane of (110) and (200) of Fe^0^ (JCPDS No. 65-4899) [40]. Furthermore, peaks occurring at 26.41, 37.65, 39.82, 40.65, 42.89, 43.76, 45.88 and 49.13° corresponded to the Bragg planes of (020), (210), (002), (201), (211), (102), (112) and (221), respectively, which were in high accordance with Fe_3_C (JCPDS No. 65-2411) [41,42]. Hence, the main components of Fe@MF-12.5-800 were Fe_3_C and Fe^0^, which is in line with the results of Gao et al. [7]. Also, the TEM images in Figure 3b,c illustrate a significant number of particles randomly dispersed within the carbonized melamine foam. Figure 3d displays the HRTEM image with a lattice spacing of 0.199 and 0.337 nm, which corresponds to the (110) crystal plane of Fe^0^ and (211) crystal plane of Fe_3_C [41,42].

### 3.3. Batch Experiment of Cr(VI) Removal

#### 3.3.1. Effect of 3D Porous Structure

Fe@MF-12.5-800 composite displays a 3D porous microstructure (Figure 2a,b), which could, in theory, facilitate the migration of contaminants and thus be beneficial for rapid Cr(VI) elimination. To demonstrate this, the Cr(VI) removal rate of the 3D porous Fe@MF-12.5-800 composite block was tested and compared with that of an identical sample ground into a powder form.

**Figure 4:**
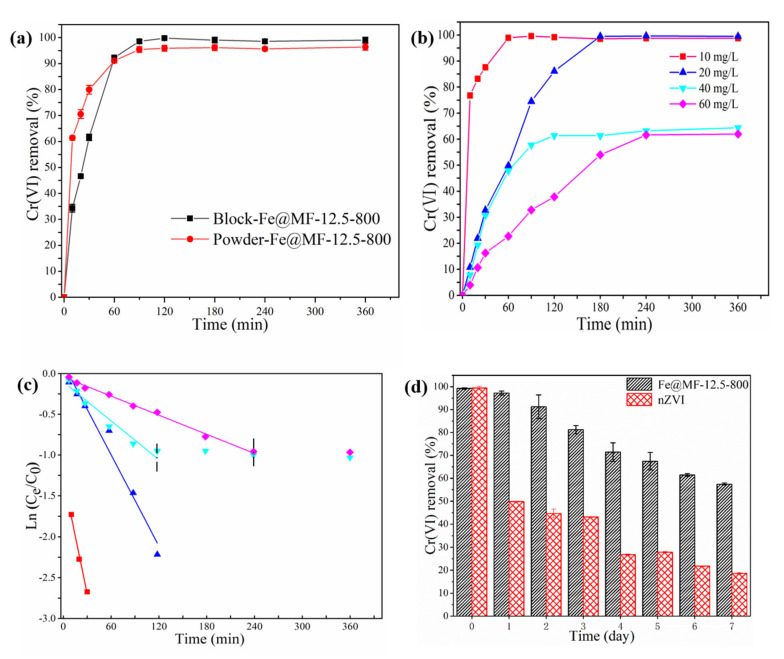
The influence of different conditions on Cr(VI) using Fe@MF-12.5-800: (**a**) effect of block and powder form; (**b**) effect of initial Cr(VI) concentration; (**c**) kinetic modeling fitted by pseudo-first-order reaction; and (**d**) the effect of aging time.

The rate of Cr(VI) removal by different Fe@MF-12.5-800 forms is shown in Figure 4a. For the initial 10 min of reaction, the Cr(VI) removal rate of the powdered Fe@MF-12.5-800 was about 2 times faster than that of the block form, which was due to the instant interaction between the Cr(VI) and powdered composite. However, the block and powdered Fe@MF-12.5-800 reached equilibrium at a similar time of about 90 min. This suggests that the large pores formed within the Fe@MF-12.5-800 framework minimized the mass transfer resistance, unlike the previous report [39]. The final Cr(VI) removal by the block sample was 99.75%, which was slightly higher than that of the powdered sample. This might be due to the partial nZVI oxidation during the grinding stage.

#### 3.3.2. Effect of Initial Cr(VI) Concentration

The influence of different Cr(VI) concentrations (10–60 mg/L) on their removal rate is presented in Figure 4b. When the concentrations were 10 and 20 mg/L, the added Fe@MF-12.5-800 could completely remove Cr(VI) present. Upon increasing the concentration to 40 and 60 mg/L, the removal rate was reduced to 64% and 62%, respectively. The fixed number of active sites in the Fe@MF-12.5-800 composite led to lower removal efficiency at higher Cr(VI) concentrations.

Previous studies have shown that ZVI reduction suits pseudo-first-order reactions [39]. Figure 4c shows that Fe@MF-12.5-800 had a good fit with the pseudo-first-order reaction, and the correlation coefficient was in the range of 0.95 to 0.99. Additionally, an increment in the initial concentration from 10 to 60 mg/L led to a reduction in the rate constant (K_obs_) from 0.047 to 0.003 min^−1^, respectively. As witnessed, the rise in the Cr(VI) concentration significantly lessened the reduction rate, probably due to the competitive effect [39]. The lower reactivity of Fe@MF-12.5-800 caused nZVI to rapidly oxidize into Fe(III) when additional Cr(VI) contacted the Fe@MF-12.5-800 surface. As a result, the K_obs_ values reduced. Furthermore, the results showed that the reaction rate was heavily influenced by the available active surface sites, which could become a constraint with increasing Cr(VI) concentration.

#### 3.3.3. Effect of the Aging Time

A decline in nZVI reactivity is typically caused by the aggregation and surface passivation of nZVI during the aging process. The adverse effects of aging have been shown in previous studies [43]. After aging nZVI anchored on biomass-activated carbon materials for one month, Zhang et al. reported that the rate of Cr removal was 30% of the initial rate [40]. As part of this study, we investigated the removal efficiency of Cr(VI) in fresh and aged Fe@MF-12.5-800 (Figure 4d). The results showed that Fe@MF-12.5-800 could remove about 60% of the Cr(VI) after a one-week aging period. In comparison, the much lower reactivity (only 20% Cr(VI) removed) of nZVI towards the target pollutant after aging was obtained, which could be linked to the partial blockage of the redox-active centers by the oxide film formed during the aging period [44]. The higher resistance of Fe@CMF-12.5-800 to air exposure could be due to the facilitated electron transfer between nZVI and carbon fiber through their contacted interface providing reduced power for Cr(VI) removal, which reduces the negative effect of passivation products formed on the nZVI surface [45].

### 3.4. Mechanisms of Cr(VI) Removal by Fe@MF-12.5-800

**Figure 5:**
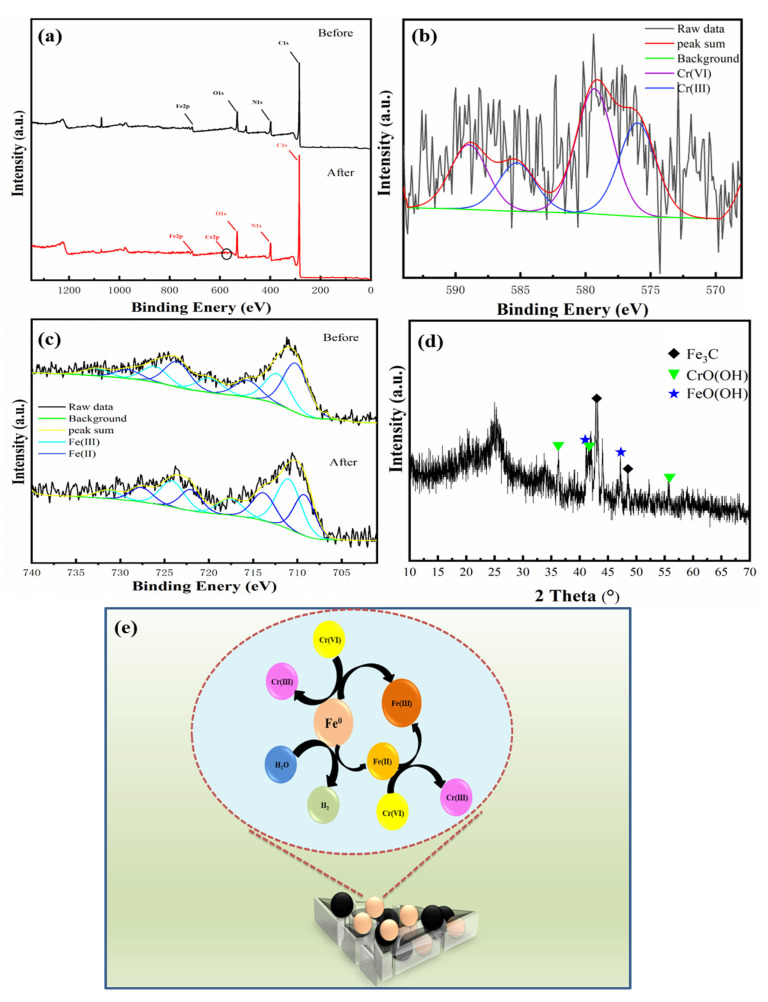
(**a**) XPS survey spectra for Fe@MF-12.5-800 before and after Cr(VI) reactions; (**b**) Cr2p XPS spectra of Fe@MF-12.5-800 after reaction; (**c**) Fe2p of Fe@MF-12.5-800 before and after reaction; (**d**) XRD of Fe@MF-12.5-800 after Cr(VI) reaction; and (**e**) plausible mechanism for monolayer Cr(VI) adsorption onto Fe@MF-12.5-800.

The reduction and adsorption mechanisms of Cr(VI) by Fe@MF-12.5-800 were analyzed using XRD and XPS. After the adsorption of Cr(VI), the small peak of Cr2p (580 eV, Figure 5a) shows the uptake of chromium on Fe@MF-12.5-800 surface. Figure 5b depicted the Cr2p XPS spectra on the surface of Fe@MF-12.5-800 after Cr (VI) removal. Two peaks located at 576.01 and 585.27 eV can be ascribed to the Cr(III) and Cr(VI), demonstrating that both Cr(VI) and Cr(III) existed [4]. 

Although, the XRD spectrum demonstrated the presence of Fe^0^ (Figure 3a), the peak at 706.7 eV ascribed to Fe^0^ before the Cr(VI) removal reaction was not clearly shown in the XPS spectrum (Figure 5c). Taking into account the surface sensitivity of XPS, the presence of the Fe^0^ peak could not be detected by XPS, which is probably due to the surface iron species being oxidized. This is also reported by Fu et al. [19]. The Fe2p peaks at 715.6 and 719.91 eV corresponded to Fe2p_3/2_ for Fe(II) and Fe(III), respectively, while the peaks at 729.08, and 732.71 eV were assigned to Fe2p_1/2_ for Fe(II) and Fe(III), respectively. Moreover, the satellite peak positions for Fe(II) were 723.58 and those for Fe(III) were 726.06 eV. The peak with binding energy located at 712.26 eV can be attributed to FeOOH.

The XRD results prove the contribution of Fe^0^ on the Cr(VI) removal as the disappearance of Fe^0^ (Figure 3a and Figure 5d) and the formation of FeO(OH) (2*θ* = 41.1°, 47.3°) and CrO(OH) (2θ = 36.4°, 41.7°, 55.9°) after the reaction. In this process, nZVI particles acted as reducing agents [19]. Inferring from the results, the equations governing the reactions can be represented as (Equations (2) and (3)):

(2) $$3{\mathrm{Fe}}^{0}+2{\mathrm{HCrO}}_{4}^{-}+14H^{+}\to 2{\mathrm{Cr}}^{3+}+3{\mathrm{Fe}}^{2+}+8H_{2}O$$

(3) $$3{\mathrm{Fe}}^{2+}+{\mathrm{HCrO}}_{4}^{-}+7H^{+}\to {\mathrm{Cr}}^{3+}+3{\mathrm{Fe}}^{3+}+4H_{2}O$$

Based on the above analysis, the Cr(VI) removal mechanism by Fe@MF-12.5-800 could be schematically described in Figure 5e. The production of Cr(III), Fe(II) and Fe(III) made the entire process environmentally friendly [5].

### 3.5. Continuous Treatment

**Figure 6:**
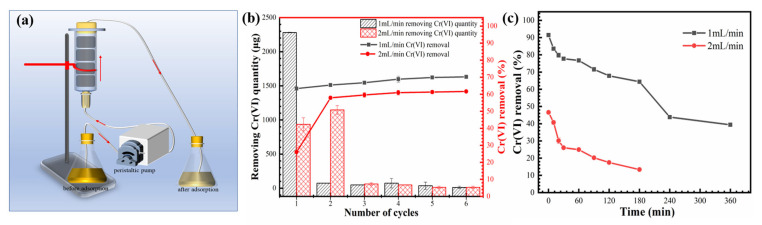
(**a**) Schematic diagram of Fe@MF-12.5-800 column migration experiment; (**b**) Cr(VI) removal efficiency for the first treatment at different flow rates; (**c**) Cr(VI) removal by Fe@MF-12.5-800 at different flow cycles.

The continuous removal of Cr(VI) through the porous Fe@MF-12.5-800 composite was tested in plastic columns. Each column was 1.6 cm in diameter and 6 cm in length, packed with 160 mg Fe@MF-12.5-800 (four pieces of identical samples). Figure 6a illustrates a schematic diagram of the reaction column set-up. In total, 360 mL of Cr(VI) heavy metal solution (10 mg/L) was continuously pumped through the reactor from the bottom at flow rates of 1 and 2 mL/min, corresponding to hydraulic retention times (HRT) of 360 and 180 min, respectively. This was followed by sampling the treated Cr(VI) solution for the remaining Cr(VI) amount. Next, the Cr(VI) solution was pumped through the reactor again to get repeated treatment.

Figure 6b depicts the continuous flow procedure for removing Cr(VI) from aqueous solutions using Fe@MF-12.5-800. When the flow rate was raised from 1 to 2 mL/min, Cr(VI) elimination decreased from 70.2% to 61.6%. Several successive cycles are shown in Figure 6c. The adsorption efficiency reduced with increasing cycles, and the Cr(VI) removed rapidly decreased at a 2 mL/min flow rate. Because of limited solute interaction time, the saturation needed for Cr(VI) removal in Fe@MF-12.5-800 reduced dramatically at higher flow rates. The chromium removal capacity of the Fe@MF-12.5-800 can be obtained by calculating the totally removed chromium and the amount of Fe@MF-12.5-800 used. Regardless of the flow rate, chromium removal capacity was about 15.67 mg/g at neutral pH (pH = 7), which is 1.96 times higher than previous published works [4].

**Figure 7:**
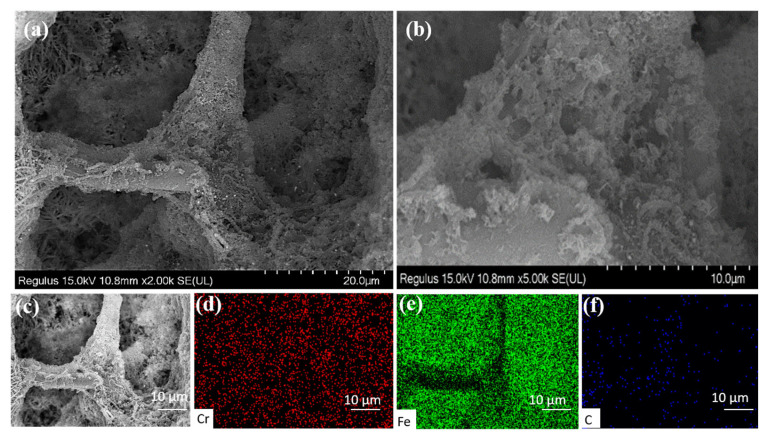
(**a**,**b**) The different magnification SEM images after reaction with Cr(VI); (**d**–**f**) EDX element mapping of Cr, Fe, and C obtained from (**c**) after reaction with Cr(VI).

The microstructure of the Fe@MF-12.5-800 composite after reacting with Cr(VI) is presented in Figure 7a,b. After the reaction, it was found that the Fe@MF-12.5-800 composites kept a typical carbonized MF structure and the framework of Fe@MF was maintained intact. The locally magnified SEM image showed laminated clusters formed on the surface of carbon fiber. These deposits are likely to be Cr_x_Fe_1-x_(OH)_3_, which is in line with previously reported works [45].

The element mapping shows evenly distributed Cr, Fe, and C elements within the framework of Fe@MF-12.5-800 (Figure 7c–f). This meant that the end products of Fe(III)/Cr(III) were finally fixed within the composite, as proven by the turbidity (OD_600_ = 0) of the effluent during the treatment. The fixation of end products would be beneficial to avoid secondary pollution, which normally occurred when the end products (e.g., Cr(III)/Fe(III)(oxy)hydroxides) were freely released into the environment [46].

## 4. Conclusions

In this study, nZVI supported by the carbonized melamine foam was examined for the effective removal of Cr(VI). Batch experiments showed that the Cr(VI) elimination efficiency increased with Fe^3+^ concentration and carbonization temperature. At pH = 7, more than 99% Cr(VI) removal was achieved. Additionally, the Cr(VI) elimination efficiency in block Fe@MF-12.5-800 was similar to its powder form. The block Fe@MF-12.5-800 is easy to separate from the environment, thus effectively reducing environmental pollution. Moreover, the simulation experiment showed that the material could be used as a filler in sewage treatment. The results proved that this approach enabled an effective and stable Cr(VI) removal in wastewater. Hence, this composite can be potentially used for heavy metal in situ repair.

## Data Availability

Data presented in this article are available at request from the corresponding author.

## Supplementary Materials

The following supporting information can be downloaded at: https://www.mdpi.com/article/10.3390/nano12111866/s1, Figure S1: (a) The synthesis process of Fe@MF (using Fe@MF-12.5-800 as an example); (b–d) photos of Fe@MF at different stages; Table S1: The content (wt.%) of each element tested by XPS and EDS; Figure S2: (a) N_2_ adsorption-desorption isotherms and (b) pore size distributions of Fe@MF-12.5-800.
