# Supplementary material for: Removal of Chromium(VI) by Nanoscale Zero-Valent Iron Supported on Melamine Carbon Foam
Source: Nanomaterials (Basel). 2022 May 30;12(11):1866. doi: 10.3390/nano12111866 (PMC9181856; doi:10.3390/nano12111866)
Supplement: Supplementary file 1 [file nanomaterials-12-01866-s001.zip › nanomaterials-1713634-supplementary.pdf]

nanomaterials

Supplementary Materials

Removal of Chromium(VI) by Nanoscale Zero-Valent Iron Supported on Melamine Carbon Foam

Qiming Li [1,†], Meili Liu [1,†], Xuchun Qiu [1], Xiang Liu [2], Malcom Frimpong Dapaah [1], Qijian Niu [3,*] and Liang Cheng [1,4,5,*]

[1] School of Environment and Safety Engineering, Jiangsu University, Zhenjiang 212013, China; jingtian19941106@163.com (Q.L.); liumeili202110525@163.com (M.L.); xuchunqiu@gmail.com (X.Q.); 5103200301@stmail.ujs.edu.cn (M.F.D.)
[2] Institute of Medicine & Chemical Engineering, Zhenjiang College, Zhenjiang 212000, China; liuxiang0222@126.com
[3] School of Agricultural Engineering, Jiangsu University, Zhenjiang 212013, China
[4] School of Civil and Mechanical Engineering, Curtin University, Perth, WA 6102, Australia
[5] Institute of Materials Engineering, Nanjing University, Nantong 226000, China
* Correspondence: niuqijian@ujs.edu.cn (Q.N.); clcheng@ujs.edu.cn (L.C.)
† These authors contributed equally to this work.

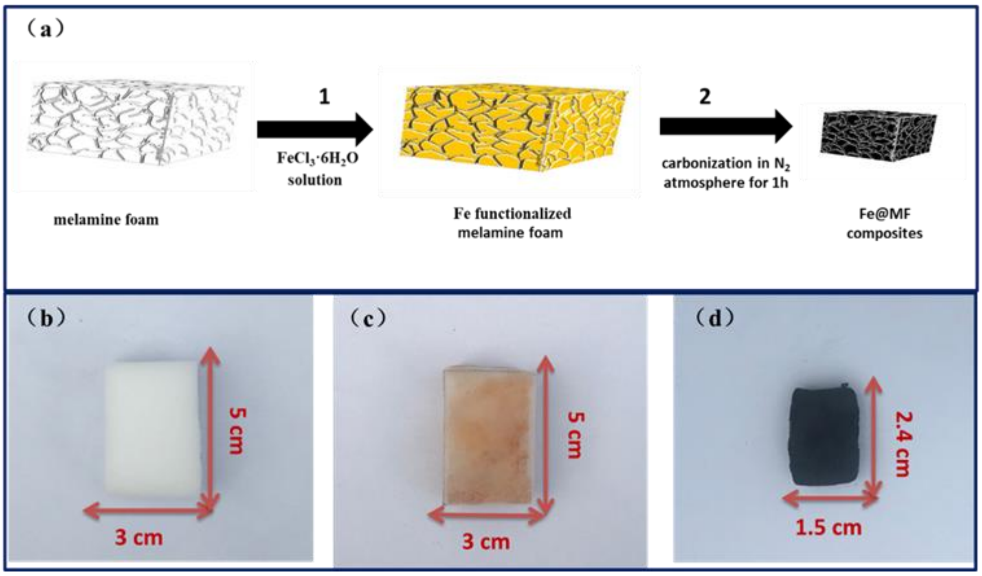

Figure S1. (**a**) The synthesis process of Fe@MF (using Fe@MF-12.5-800 as an example); (**b**–**d**) photos of Fe@MF at different stages.

Table S1. The content (wt. %) of each element tested by XPS and EDS.

Element	XPS Mass Percentage (%)	Error	EDS Mass Percentage (%)
Fe	16.22	1.66	14.13
C	60.11	1.73	55.16
N	5.76	0.91	6.08
O	17.82	1.00	24.13
Cr	0.09	1.22	0

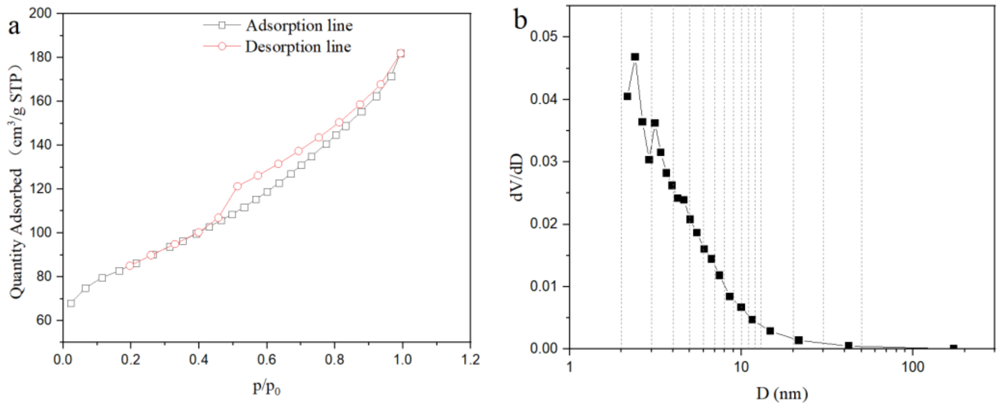

Figure S2. (**a**) N_2 adsorption-desorption isotherms and (**b**) pore size distributions of Fe@MF-12.5-800.
